# Carrier-mediated reduction mechanism in WO_3_ nanowires under electron-beam irradiation

**DOI:** 10.1093/jmicro/dfaf058

**Published:** 2026-01-02

**Authors:** Sho Nekita, Itsuki Misono, Kazuhiro Yasuda, Yusuke Shimada, Jyh-Tyng Chou, Tetsuya Okuyama, Satoshi Hata

**Affiliations:** Interdisciplinary Graduate School of Engineering Sciences, Kyushu University, 6-1 Kasugakoen, Kasuga, Fukuoka 816-8580, Japan; Interdisciplinary Graduate School of Engineering Sciences, Kyushu University, 6-1 Kasugakoen, Kasuga, Fukuoka 816-8580, Japan; Faculty of Engineering Department of Applied Quantum Physics and Nuclear Engineering, Kyushu University, 744 Motooka, Nishi, Fukuoka 819-0395, Japan; The Ultramicroscopy Research Center, Kyushu University, 744 Motooka, Nishi, Fukuoka 819-0395, Japan; Faculty of Engineering Sciences, Kyushu University, 6-1 Kasugakoen, Kasuga, Fukuoka 816-8580, Japan; National Institute of Technology, Kurume College, 1-1-1 Komorino, Kurume, Fukuoka 830-8555, Japan; Faculty of Engineering Sciences, Kyushu University, 6-1 Kasugakoen, Kasuga, Fukuoka 816-8580, Japan; The Ultramicroscopy Research Center, Kyushu University, 744 Motooka, Nishi, Fukuoka 819-0395, Japan; Faculty of Engineering Sciences, Kyushu University, 6-1 Kasugakoen, Kasuga, Fukuoka 816-8580, Japan

**Keywords:** electron-beam irradiation, carrier-mediated reduction, electron–hole dynamics, electron energy-loss spectroscopy, tungsten oxide, nanowires

## Abstract

Electron-beam irradiation often induces unintended structural and chemical changes in materials. Here, we show that damage and reduction in tungsten trioxide (WO_3_) nanowires are primarily driven by a carrier-mediated ionization process. *In situ* electron microscopy and electron energy-loss spectroscopy reveal structural degradation accompanied by the reduction of W^6+^ to W^5+^, while carrier dynamics simulations identify persistent, high-density electron–hole populations. Quantitative analyses and control experiments indicate that knock-on displacement and heating contribute minimally. This study establishes a microscopy-based quantitative framework for understanding electron-beam-induced damage and redox processes, highlighting the potential of electron microscopy for mechanistic insights and nanoscale chemical patterning in oxides.

## Teaser

We quantitatively identify carrier-mediated ionization as the dominant mechanism of electron-beam-induced reduction in WO₃ nanowires. In situ STEM–EELS observations and carrier-transport simulations reveal persistent high-density carriers that drive W^6+^→W^5+^ conversion. These findings clarify beam–matter interactions in reducible oxides and improve microscopy of beam-sensitive materials.

Electron microscopy is an essential tool for characterizing materials at the nanoscale [1,2]. However, exposure to electron beams can induce material damage [3–8]. While such beam-induced structural and chemical changes complicate analysis, they can also be exploited for controlled material processing, particularly in metal oxides [9,10]. Electron-beam-induced damage is generally attributed to three primary mechanisms: knock-on, ionization and local heating. Knock-on damage arises when incident electrons transfer sufficient kinetic energy to displace atoms from their lattice sites [3,4]. Ionization damage or radiolysis results from electronic excitations [6,7], whereas local heating originates from inelastic scattering processes [8].

Among metal oxides, tungsten trioxide (WO_3_) is a prototypical reducible material extensively studied for its electrochromic [11], gas-sensing [12] and catalytic properties [13]. Despite considerable research, the mechanism of electron-beam-induced damage and reduction in WO_3_—particularly the quantitative pathway by which excited carriers drive valence changes and oxygen loss—remains poorly understood [6,7]. WO_3_ nanowires, with their high aspect ratios and surface-sensitive transport, are especially susceptible to localized structural or chemical changes, which can directly affect their electrical and sensing properties [12], highlighting the importance of understanding their beam-induced response. Here, we address this knowledge gap by investigating the three primary mechanisms of beam-induced damage—knock-on displacement, ionization and local heating—using combined experimental and theoretical approaches. Through *in situ* electron energy-loss spectroscopy (EELS), low-voltage scanning electron microscopy (SEM), vacuum annealing experiments and numerical simulations, we demonstrate that electron-beam-induced chemical reduction in WO_3_ is predominantly governed by a carrier-mediated ionization mechanism.

WO_3_ nanowires were synthesized via a hydrothermal method following a previously reported procedure [12]. Scanning transmission electron microscopy (STEM) with annular dark-field (ADF) imaging, bright-field (BF) imaging and EELS analyses were performed using a Titan Cubed G2 60–300 (Thermo Fisher Scientific) equipped with a Gatan Imaging Filter Quantum 965 spectrometer (Gatan Inc.). Accelerating voltages of 300 and 80 kV were used, and the probe current was maintained at 110 pA for all measurements. STEM–ADF images were acquired with a convergence semi-angle of 17.9 mrad and a collection semi-angle range of 52–200 mrad. STEM–BF images were obtained using the same convergence semi-angle (17.9 mrad) and a collection semi-angle range of 0–29 mrad. Sequential EELS spectra were collected from the same region with an energy dispersion of 0.05 eV per channel and an energy resolution of approximately 1.1 eV. Core-loss edges were extracted by subtracting the background using power-law fitting. Low-voltage SEM observations were conducted with a field-emission SEM (Ultra, Carl Zeiss) at an acceleration voltage of 2 kV and a probe current of 0.5 nA. Vacuum annealing experiments were performed using a dedicated heating holder (EM-31670, JEOL Ltd), in which WO_3_ nanowires were heated to 623 K for 20 min under high vacuum (∼10^−5 ^Pa). Conventional transmission electron microscopy (TEM) images were acquired before and after annealing using a JEM-2100Plus (JEOL Ltd) operated at 200 kV.

**Fig. 1.**
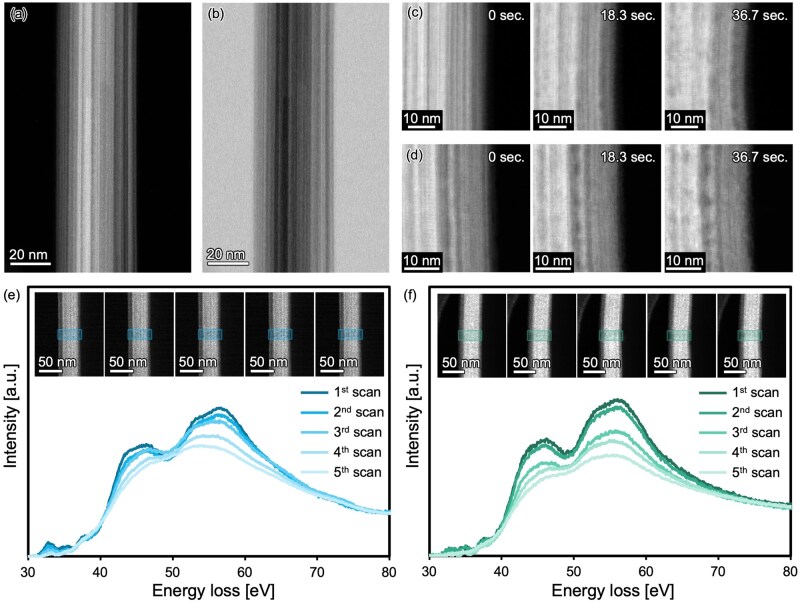
(a) STEM–ADF and (b) STEM–BF images of the same region of a WO_3_ nanowire acquired prior to electron-beam irradiation; the absence of surface contamination or amorphous layers is confirmed by the consistent contrast between the two imaging modes. (c and d) Sequential STEM–ADF images of WO_3_ nanowires under continuous EBI at (c) 300 kV and (d) 80 kV; the progressive darkening and contrast changes indicate beam-induced structural degradation. (e and f) Sequential EELS spectra acquired from the irradiated regions shown in the insets under continuous electron-beam exposure at (e) 300 kV and (f) 80 kV; the W–O_2,3_ edge gradually broadened and shifted toward lower energy losses as the number of acquisitions increased, indicating a reduction of W^6+^ to W^5+^.

Prior to electron-beam irradiation (EBI), STEM–ADF and STEM–BF images acquired from the same region confirmed the absence of surface contamination or amorphous layers as shown in Fig. 1a and b, ensuring that the subsequent observations reflect intrinsic beam-induced structural and chemical changes. EBI was applied to the synthesized WO_3_ nanowires to investigate beam-induced structural and chemical changes. STEM–ADF time-series images acquired at accelerating voltages of 300 and 80 kV are shown in Fig. 1c and d, respectively. During the initial irradiation stage, well-defined atomic-column contrast was observed at both voltages. With continued exposure, the atomic-column contrast gradually diminished, lattice fringes near the surface became blurred, and the nanowire volume progressively decreased. This degradation behavior was consistently observed across multiple nanowires under identical EBI conditions.

Corresponding changes in the electronic structure under EBI were revealed by EELS measurements (Fig. 1e and 1f). With increasing acquisition cycles, the W–O_2,3_ edge (W 5p → 5d transition) showed pronounced peak broadening, an overall intensity decrease, and a systematic shift toward lower energy-loss values at both 300 and 80 kV. These spectral changes are characteristic of tungsten reduction in WO_3_. In the octahedral coordination of WO_3_, the W 5d orbitals are split into t_2g_ and e_g_ states by the crystal field [14]. Continued reduction induces octahedral distortion and local disorder, which relaxes the orbital splitting and broadens the O_2,3_ edge. Although the W^6+^ 5d orbitals are nominally unoccupied, those of the reduced species (W^5+^ and W^4+^) are partially filled, reducing the transition probability and leading to decreased spectral intensity. The characteristic shift of the O_2,3_ edge toward lower energy-loss values is consistent with previously reported EELS features of WO_3_-based oxides containing mixed valence states (W^6+^, W^5+^, and W^4+^) [15]. These EELS changes cannot be attributed to surface contamination or simple mass–thickness variations, confirming that the observed evolution arises from a reduction process. In the following section, the possible driving mechanisms of this beam-induced damage and reduction—knock-on displacement, ionization, and local heating—are quantitatively analyzed and compared.

**Fig. 2.**
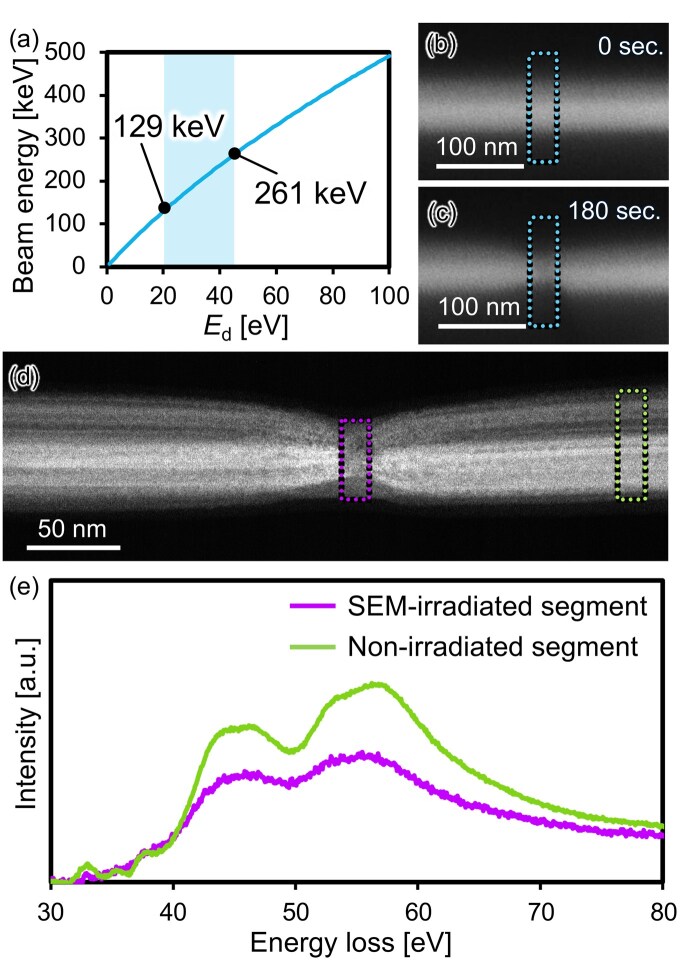
(a) Calculated relationship between the displacement threshold energy *E*_d_ = 20–45 eV and the corresponding knock-on threshold electron energy; the shaded region represents the reported range of *E*_d_ for oxygen atoms in metal oxides. (b and c) SEM images of a WO_3_ nanowire before and after EBI at 2 kV with a probe current of 0.5 nA for 180 s; the dashed rectangle indicates the region irradiated by a scanned electron beam. (d) STEM–ADF image acquired after EBI, showing noticeable structural changes in the irradiated region; although the 2-kV beam energy is substantially below the knock-on threshold (129–261 keV), morphological changes are still observed, indicating that the beam-induced damage in WO_3_ cannot be explained solely by the knock-on effect. (e) STEM–EELS spectra acquired from the EBI and non-EBI regions, showing a decrease in the W–O_2,3_ edge intensity in the irradiated area, consistent with partial reduction from W^6+^ to W^5+^.

The contribution of knock-on damage was quantitatively evaluated to assess elastic scattering. The maximum kinetic energy *E*_max_ transferable from an incident electron to a lattice atom is given by the relativistic momentum conservation law [4]:


(1) $$E_{\text{max}}=\frac{2E\left(E+2m_{0}c^{2}\right)}{Mc^{2}},$$

where *E* denotes the electron kinetic energy, *m*_0_ denotes the electron rest mass, *M* denotes the target atomic mass, and *c* denotes the speed of light. For oxygen atoms in oxides, the displacement threshold energy *E*_d_ has been reported to be 20–45 eV [16,17]. The corresponding electron kinetic energy required to exceed this threshold is approximately 129–261 keV (Fig. 2a). Although 300-kV electrons can in principle displace oxygen, displacement per atom (dpa) calculations with use of SMOTT/POLY code [18] based on Mott cross sections show that the STEM–ADF conditions in Fig. 1c (current density *j *= 6.9 × 10^8^ A m^−2^, dwell time 5.0 μs) induce limited displacement damage in the range of 3.1 × 10^−6^ to 4.2 × 10^−5^ (dpa/scan) under an assumption of *E*_d_ values of 20–45 eV. Therefore, even though 300-kV electrons can, in principle, supply sufficient kinetic energy, knock-on displacement cannot be the primary origin of the structural degradation observed in our experi-ments. The comparable structural degradation observed even at 80 kV (Fig. 1d) further supports this conclusion. Similar contrast variations and volume shrinkage were also observed under 2-kV EBI in SEM (Fig. 2b–d), where the electron energy is several orders of magnitude below the knock-on threshold. Together, these observations indicate that elastic displacement of atoms is not the predominant mechanism driving beam-induced structural degradation. Moreover, STEM–EELS measurements performed after 2-kV irradiation consistently revealed a decrease in the W–O_2,3_ edge intensity as shown in Fig. 2e, indicating partial reduction from W^6+^ to W^5+^. This change is similar to that observed in the in situ 80-kV and 300-kV STEM–EELS measurements, suggesting that the structural degradation induced by 2-kV electrons proceeds via the same underlying mechanism as that observed at higher accelerating voltages.

**Fig. 3.**
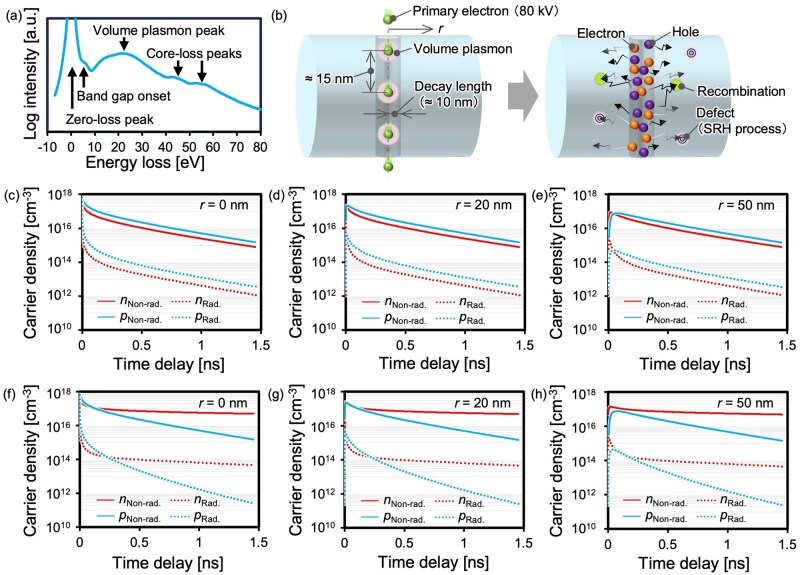
(a) EELS spectrum of WO_3_ nanowires; the spectrum exhibits a strong volume-plasmon peak at approximately 23 eV, accompanied by core-loss peaks at higher energies and a band-gap onset below 3 eV. (b) Schematic of carrier generation and recombination processes in WO_3_ nanowires under electron irradiation; a primary electron (80 kV) excites volume plasmons, which decay within a length of ∼10 nm and produce electron–hole pairs, with an average interval of ∼15 nm. The generated carriers diffuse and decay through SRH and radiative recombination processes. (c–h) Calculated temporal evolution of electron and hole densities at different radial positions (*r *= 0–50 nm) within WO_3_ nanowires. The results for *k*_1__*e*_ = *k*_1__*h*_ and *k*_1__*e*_ = 0.1 *k*_1__*h*_ are shown in (c–e) and (f–h), respectively. Solid and dashed lines represent results for the nonradiative and radiative recombination models, respectively.

Ionization damage primarily arises from inelastic interactions between incident electrons and the target material. As shown in Fig. 3a, the EELS spectrum of WO_3_ nanowires exhibits a pronounced volume-plasmon peak at ∼23 eV, indicating that volume-plasmon excitation is the dominant inelastic scattering process in WO_3_. In comparison, the onset associated with direct valence-electron excitation is much weaker, suggesting that its contribution to the generation of electron–hole pairs is minor relative to plasmon-mediated processes. The kinetic energy loss of incident electrons during inelastic scattering [19] yields energy-loss rates of approximately 1.5 and 0.5 eV nm^−1^ at 80 and 300 kV, respectively (see Supplementary Data A). Assuming, as an upper-limit approximation, that all inelastic scattering events correspond to plasmon excitation, the average distance between successive plasmon excitations is estimated to be approximately 15 nm at 80-kV irradiation. In the following analysis, we focus on the 80-kV irradiation as a representative case.

Each plasmon decays on a femtosecond timescale, generating multiple electron–hole pairs through energy partitioning [20,21]. A single volume plasmon can produce up to three electron–hole pairs [21]. Assuming a plasmon-decay length of 10 nm [22], the local carrier density per excitation event is estimated to reach ∼10^17^ cm^−3^, several orders of magnitude higher than the reported defect density in WO_3_ (∼10^11^ cm^−3^) [23].

To quantify the carrier dynamics (Fig. 3b), the time evolutions of electron and hole densities, denoted by *n*(*r*, *t*) and *p*(*r*, *t*), respectively, were simulated by solving the coupled diffusion–recombination equations in cylindrical coordinates using a finite-difference method [24]. We use a model incorporating the Shockley–Read–Hall (SRH) process and Langevin-type recombination [25,26]:


(2) $$\frac{\partial n}{\partial t}\;=\;{-k}_{1e}n-k_{L}np+D_{e}[\frac{d^{2}n}{dr^{2}}+\frac{1}{r}\frac{dn}{dr}],$$

(3) $$\frac{\partial p}{\partial t}\;=\;{-k}_{1h}p-k_{L}np+D_{h}[\frac{d^{2}p}{dr^{2}}+\frac{1}{r}\frac{dp}{dr}],$$

where *k*_1__*e*_ and *k*_1__*h*_ denote the SRH recombination rate constants for electrons and holes, respectively, *k_L_* denotes the Langevin-type radiative recombination coefficient, and *D_e_* and *D_h_* are the diffusion coefficients for electrons and holes, respectively; *k_L_* is expressed in terms of the electron and hole mobi-lities (*µ_e_*, *µ_h_*) and the dielectric constant (*ε*) as follows [25]:


(4) $$k_{L}=q\frac{\mu_{e}+\mu_{h}}{\varepsilon }.$$

Because Langevin-type radiative recombination has been reported to overestimate the decay of excited carriers in inorganic semiconductors [26], a nonradiative model excluding the radiative recombination term was also considered for comparison. For a 110-pA electron probe, electrons arrive at an average interval of ∼1.46 ns. The upper time limit for the calculation was set to 1.46 ns, and the residual carrier density after this period was incorporated into the subsequent generation term produced by the next incident electrons. By substituting the material parameters of WO_3_ (*D_e_* = 0.57 cm^2^ s^−1^, *D_h_* = 0.127 cm^2^ s^−1^, *k*_1__*h*_ = 2.27 × 10^−9^ s^−1^, *m_e_* = 21.99 cm^2^ V^−1^ s^−1^, *m_h_* = 4.92 cm^2^ V^−1^ s^−1^, *e *= 1.57 × 10^−13^ F cm^−1^) [23,27], the cumulative carrier contribution reached a steady state after several iterations. Details of the numerical procedure are presented in Supplementary Data B.

Simulations showed that, when the electron SRH recombination rate was assumed equal to that of holes (*k*_1__*e*_ = *k*_1__*h*_), the carrier density near the nanowire center peaked at ∼10^17^ cm^−3^ immediately after excitation and remained in the range of 10^12^–10^15^ cm^−3^ before the next electron arrival (Fig. 3c–e). As WO_3_ is a typical n-type semiconductor, donor levels lie near the conduction band, making electron trapping less frequent than hole trapping. When the electron recombination rate was assumed much slower (*k*_1__*e*_ = 0.1*k*_1__*h*_), the electron density increased by one to two orders of magnitude, while the hole density decreased correspondingly (Fig. 3f–h). This enhanced retention of quasi-steady electrons under *n*-type conditions effectively increases the supply of electrons available for the W^6+^→W^5+^ reduction process. At a higher accelerating voltage (300 kV), both carrier densities decreased by approximately one order of magnitude (see Supplementary Data C).

Under all conditions, both electron and hole densities substantially exceed the reported defect density in WO_3_ nanowires (∼10^11^ cm^−3^) [23], indicating that defect levels can be readily saturated. Such high-density electronic excitation persists over time, producing a quasi-steady occupation of the W 5d conduction-band minimum, corresponding to W^(6−^^*x*^^)+^ states. Concurrently, a high hole density accumulates at the O 2p valence-band maximum, creating a local electron deficiency. Hole localization in the O 2p orbitals is expected to weaken W–O bonds and promote oxygen desorption, thereby driving the chemical reduction process. This ionization-driven mechanism consistently accounts for the observed W^6+^→W^5+^ conversion and the similar damage behavior observed at 80 and 300 kV.

The contribution of beam-induced heating was quantitatively evaluated using a steady-state heat-diffusion model that accounts for axial conduction along the nanowires (see Supplementary Data D) [28,29]. The calculated temperature rise (Δ*T*) under typical STEM–ADF imaging conditions was below 10 K, indicating that thermal effects are negligible compared with ionization-driven processes. Including radiative heat loss based on the Stefan–Boltzmann law [30] (emissivity ≈ 0.72) [31] is expected to further reduce Δ*T*. Although transient local heating may occur near the beam center, the energy dissipation time (<10^−9^ s) is shorter than the electron interarrival interval (10^−9^–10^−8^ s), preventing the development of a significant temperature gradient.

**Fig. 4.**
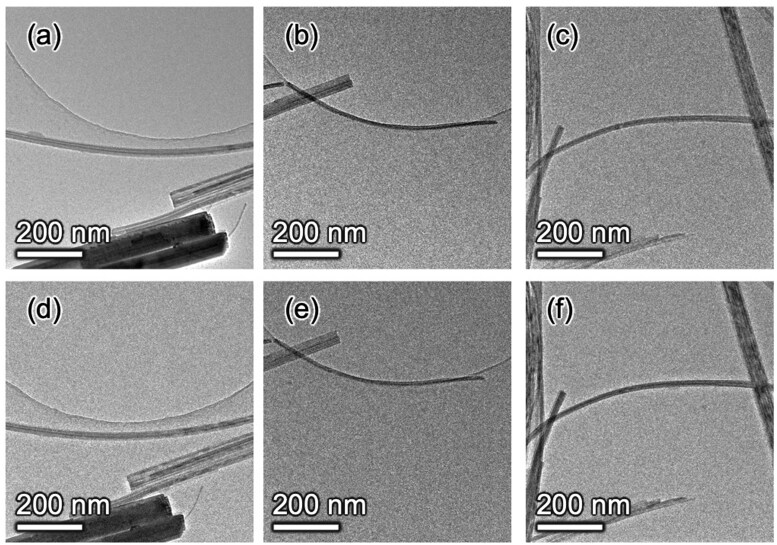
TEM images of WO_3_ nanowires before (a–c) and after (d–f) vacuum annealing at 623 K for 20 min. No apparent morphological changes or degradation of crystallinity were observed.

To experimentally confirm the negligible role of beam-induced heating, vacuum annealing was performed at 623 K and ∼10^−5 ^Pa for 20 min. This temperature was chosen to be substantially higher than the maximum Δ*T* predicted from calculations (≤10 K), ensuring that any purely thermal effects would be observable. As shown in Fig. 4, no detectable morphological or contrast changes were observed after annealing. These results confirm that heating alone does not cause noticeable structural degradation under the present conditions. Therefore, local heating can be ruled out as a dominant mechanism for beam-induced structural changes, further supporting the ionization-driven, carrier-mediated reduction model described above.

In summary, we quantitatively elucidated the mechanisms of electron-beam-induced degradation in WO_3_ nanowires. Our analyses demonstrate that the observed structural degradation and accompanying tungsten valence reduction are not due to knock-on displacement or local heating, but are driven by carrier-mediated ionization processes. These findings resolve a long-standing ambiguity regarding oxide beam-induced damage and provide a quantitative framework for understanding and predicting ionization-driven reduction in reducible oxides. While ionization-induced damage cannot be fully avoided, it can be substantially reduced by minimizing the number of incident electrons, for example, by lowering the probe current. For WO_3_ nanowires synthesized under identical conditions, an ultra-low probe current of 2.3 pA caused no detectable structural or chemical changes [32], demonstrating that low-dose STEM operation is an effective means of suppressing beam-induced artifacts. Furthermore, this study offers valuable insights into how beam-induced carrier dynamics influence chemical changes in oxide nanostructures.

## Supplementary Material

dfaf058_Supplementary_Data
