# Supplementary material for: Carrier-mediated reduction mechanism in WO3 nanowires under electron-beam irradiation
Source: Microscopy (Oxf). 2026 Jan 2;75(3):294–300. doi: 10.1093/jmicro/dfaf058 (PMC13280754; doi:10.1093/jmicro/dfaf058)
Supplement: dfaf058_Supplementary_Data [file dfaf058_supplementary_data.zip › dfaf058_Supplementary_Data/Microscopy_Supp_dfaf058.docx]

***Supplementary Data for***

**Carrier-mediated reduction mechanism in WO_3_ nanowires under electron-beam irradiation**

Sho Nekita^1^*, Itsuki Misono^1^, Kazuhiro Yasuda^2,3^, Yusuke Shimada^4^, Chou Jyh-Tyng^5^, Tetsuya Okuyama^4^, and Satoshi Hata^3,4^

^1^Interdisciplinary Graduate School of Engineering Sciences, Kyushu University, 6-1 Kasugakoen, Kasuga, Fukuoka 816-8580, Japan

^2^Faculty of Engineering Department of Applied Quantum Physics and Nuclear Engineering, Kyushu University, 744 Motooka, Nishi, Fukuoka 819-0395, Japan

^3^The Ultramicroscopy Research Center, Kyushu University, 744 Motooka, Nishi, Fukuoka 819-0395, Japan

^4^Faculty of Engineering Sciences, Kyushu University, 6-1 Kasugakoen, Kasuga, Fukuoka 816-8580, Japan

^5^National Institute of Technology, Kurume College, 1-1-1 Komorino, Kurume, Fukuoka 830-8555, Japan

*Corresponding author

Phone: +81-092-583-7580

E-mail: nekita.sho.694@s.kyushu-u.ac.jp

1. **Calculation of Energy Loss based on Bethe’s Formula**
2. **Calculation of Carrier Dynamics in a WO_3_ Nanowire**
3. **Carrier Density and Temporal Evolution under 300 kV Irradiation Condition**
4. **Calculation of Beam-Induced Temperature Rise**
5. **Calculation of Energy Loss based on Bethe’s Formula**

The energy loss of incident electrons per unit path length was calculated using Bethe’s formula^[S1]^:

$\frac{\text{dE}}{\text{dL}}\text{[eV }\text{Å}^{\text{-1}}\text{] = 785}\frac{\text{ρZ}}{\text{A}\text{E}_{\text{e}}}\text{ln}\left[ \frac{\text{1.166}\text{E}_{\text{e}}}{\text{E}_{\text{i}}} \right]$, (S-1)

where *A* is the atomic weight, *ρ* is the density [g cm^-3^], *E_e_* is the kinetic energy of the incident electron [eV], and *E_i_* is the mean ionization energy [eV], which is given by

$E_{i}\text{ = 9.76}\text{Z}\text{ +}\frac{\text{58.5}}{\text{Z}^{\text{0.19}}}\text{ (for }\text{Z}\text{ ≥ 13) }\text{.}$ (S-2)

Using the material parameters for WO₃ (*ρ* = 6.28 g cm^-3^, *Z*_eff_ = 24.5, *A*_eff_ = 57.96 g mol^-1^), the calculated energy-loss rates were approximately 1.5 eV nm^-1^ at 80 kV and 0.5 eV nm^-1^ at 300 kV.

**B. Calculation of Carrier Dynamics in a WO_3_ Nanowire**

The time evolution of electron and hole densities was numerically solved according to Eqs. (2)–(4) in the main text under the following finite-difference approximation in cylindrical coordinates:

$n_{i,j}=n_{i-1,j}-dt(k_{1e}n_{i-1,j}+k_{L}n_{i-1,j}p_{i-1,j}$)

$+D_{e}dt\left[ \frac{n_{i-1,j+1}+n_{i-1,j-1}-2n_{i-1,j}}{{dr}^{2}}-\frac{1}{r_{j}dr}\frac{n_{i-1,j+1}-n_{i-1,j-1}}{dr} \right]$, (S-3)

$p_{i,j}=p_{i-1,j}-dt(k_{1h}p_{i-1,j}+k_{L}n_{i-1,j}p_{i-1,j}$)

$+D_{h}dt\left[ \frac{p_{i-1,j+1}+p_{i-1,j-1}-2p_{i-1,j}}{{dr}^{2}}-\frac{1}{r_{j}dr}\frac{p_{i-1,j+1}-p_{i-1,j-1}}{dr} \right]$, (S-4)

where *i* and *j* are the indices along the time and radial axes, respectively. The axisymmetric boundary condition ensures *n_i,j_* = *n_i,-j_* and *p_i,j_* = *p_i,-j_*. At *r* = 0, the derivative $\frac{\partial n}{\partial r}$ = $\frac{\partial n}{\partial r}$ = 0 was imposed.

A uniform spatial mesh of $\Delta$*r* =10 nm and a temporal step of $\Delta$*t* = 5.0×10^-14^ s were used. Each irradiation event was simulated for *t*_max_ =1.46 ns, corresponding to the average arrival interval between individual electrons at a beam current of 110 pA. The material parameters of WO_3_ adopted in the calculation are:

*D_e_* = 0.57 cm^2^ s^-1^,

*D_h_* = 0.127 cm^2^ s^-1^,

*k*_1_*_h_* = 2.27 × 10^-9^ s^-1^,

*μ_e_* = 21.99 cm^2^ V^-1^ sec^-1^,

*μ_h_* = 4.92 cm^2^ V^-1^ sec^-1^, and

*ε* = 1.57 × 10^-13^ F cm^-1^.

The SRH recombination rate constant for electrons *k*_1_*_e_* was varied under two conditions: *k*_1_*_e_* = *k*_1_*_h_* as the upper limit and *k*_1_*_e_* = 0.1 *k*_1_*_h_* as a markedly lower value. The Langevin recombination coefficient was calculated as $\text{k}_{\text{L}}\text{=}\text{q}\frac{\text{μ}_{\text{e}}\text{+}\text{μ}_{\text{h}}}{\text{ε}}$ for the radiative model, while *k_L_* = 0 was used for the non-radiative model.

At *t* = 0, a uniform carrier excitation was introduced within a cylindrical region of radius *r*_0_ = 10 nm, corresponding to the decay length of the volume plasmon excitation. The initial carrier densities were set to *n*_0_ = *p*_0_ = 6.3×10^17^ cm^-3^ inside this region. Outside the excitation volume, the electron density was initialized to the background defect density of 7.6 ×10^11^ cm^-3^, representing the intrinsic donor concentration in WO_3_ nanowires, whereas the hole density was set to zero.

To reproduce continuous beam irradiation, the simulation was repeated by adding the residual carrier distribution at the end of one event (*t* =1.46 ns) to the newly generated carriers at *t* = 0. After several iterations, the carrier distribution reached a quasi-steady state. The convergence criterion was defined as a relative change of less than 5 % in the carrier densities between successive iterations.

The results for the equal recombination rate condition (*k*_1_*_e_* = *k*_1_*_h_*) and the lower electron recombination rate condition (*k*_1_*_e_* = 0.1*k*_1_*_h_*) are shown in Figures S1 and S2, respectively. As shown in Figure S1, steady-state convergence was achieved after three iterations for both the radiative and non-radiative models in the case of *k*_1_*_e_* = *k*_1_*_h_*. As shown in Figure S2, in the case of *k*_1_*_e_* = 0.1*k*_1_*_h_*, the carrier distributions converged within 4 iterations for the radiative model and 12 iterations for the non-radiative model, with the relative change in carrier density reduced to within 5 %.


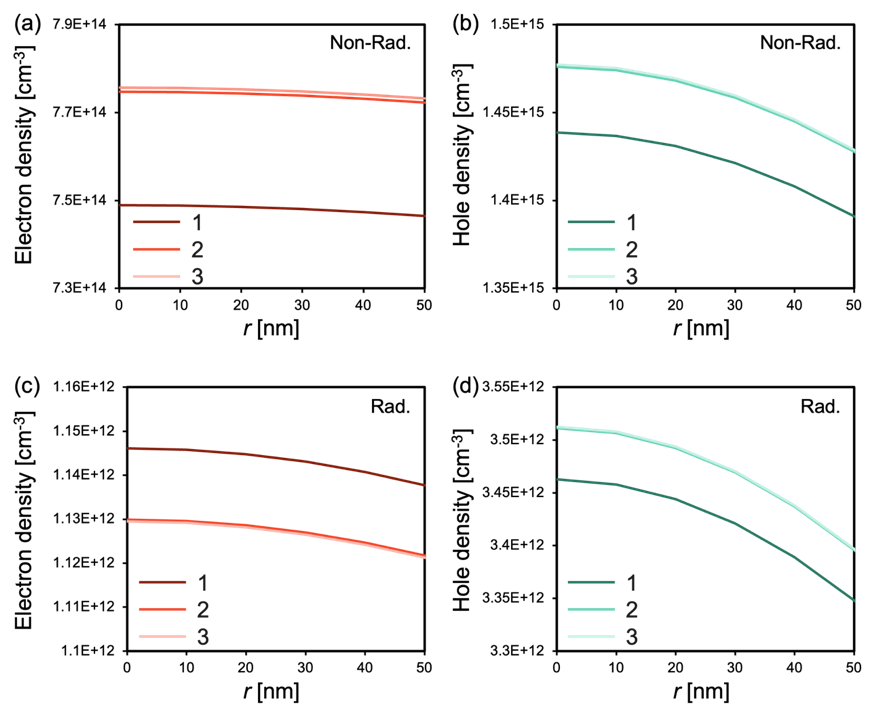


**Figure S1.** Radial distributions of (a, c) electron and (b, d) hole densities at *t* = 1.46 ns obtained from the iterative simulations for (a, b) the non-radiative model and (c, d) the radiative model in the case of *k*_1_*_e_* = *k*_1_*_h_*. The numbers (1–3) indicate the iteration steps, where each iteration corresponds to one irradiation cycle. The carrier distributions converge within three iterations, satisfying the convergence criterion of less than 5% relative change between successive iterations for both models.


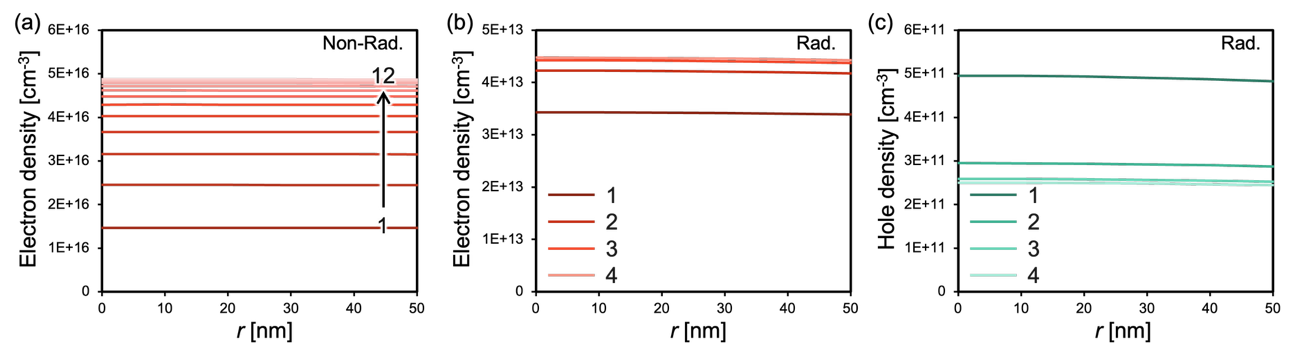


**Figure S2.** Radial distributions of (a, b) electron and (c) hole densities at *t* = 1.46 ns obtained from the iterative simulations for (a) the non-radiative model and (b, c) the radiative model in the case of *k*_1_*_e_* = 0.1*k*_1_*_h_*. The numbers (1–12 and 1–4) indicate the iteration steps, where each iteration corresponds to one irradiation cycle. The carrier distributions converge within three iterations, satisfying the convergence criterion of less than 5% relative change between successive iterations for both models.

**C. Carrier Density and Temporal Evolution under 300 kV Irradiation Condition**

To examine the influence of accelerating voltage on carrier dynamics, the time evolution of electron and hole densities were simulated for the 300 kV irradiation condition using the same diffusion–recombination model described in Supplementary Data B. Two representative cases were considered:

(1) the electron SRH recombination rate constant equal to that of holes (*k*_1_*_e_* = *k*_1_*_h_*)

(2) a slower electron recombination rate (*k*_1_*_e_* = 0.1*k*_1_*_h_*).

Both the radiative (Rad.) and non-radiative (Non-Rad.) recombination models were evaluated to assess the role of carrier decay pathways. As shown in Figure S3, the overall temporal behavior at 300 kV is qualitatively like that at 80 kV (see Main Text, Fig. 3(c–h)), exhibiting rapid decay immediately after excitation followed by a slower recombination-limited tail. However, the absolute carrier densities are approximately one order of magnitude lower, reflecting the smaller inelastic energy-loss rate at 300 kV ( ~0.5 eV nm^-1^).


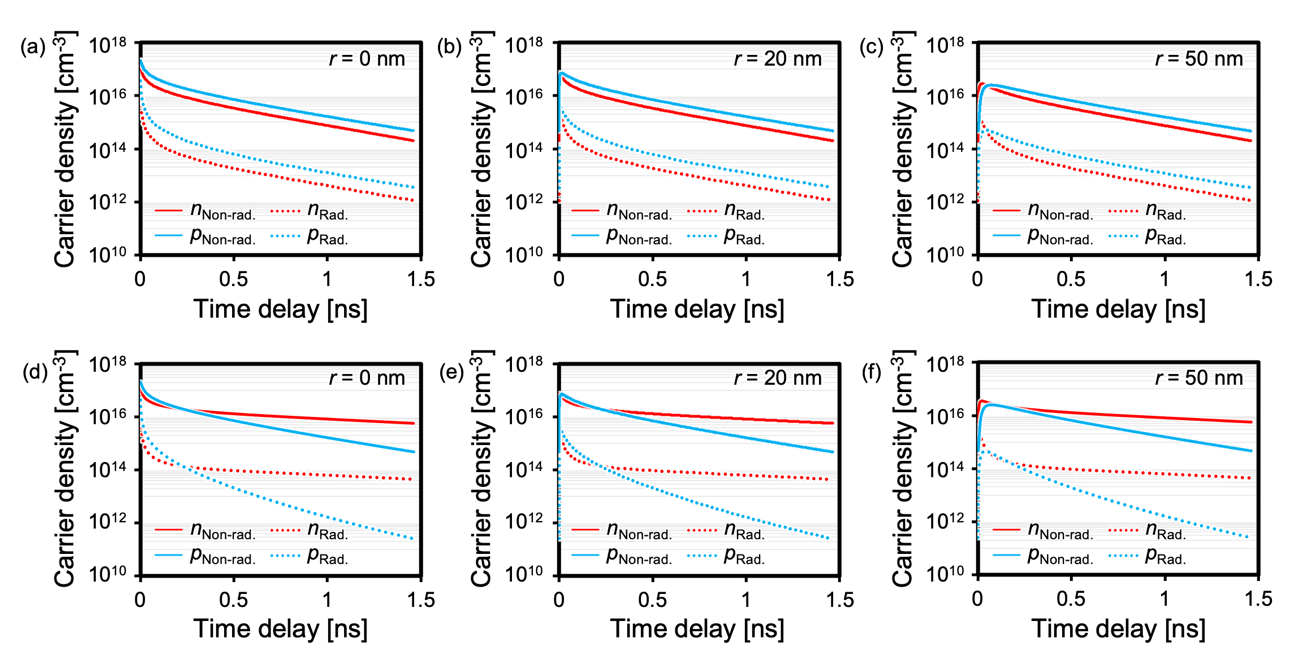


**Figure S3.** Calculated temporal evolution of electron and hole densities at different radial positions (*r* = 0–50 nm) within the WO_3_ nanowire. The results for *k*_1_*_e_* = *k*_1_*_h_* and *k*_1_*_e_* = 0.1 *k*_1_*_h_* are shown in (a–c) and (d–f), respectively. Solid and dashed lines represent results for non-radiative and radiative recombination models, respectively.

1. **Calculation of Beam-Induced Temperature Rise**

The temperature rise (Δ*T*) caused by electron-beam irradiation was quantitatively evaluated to assess the possible contribution of beam-induced heating. The deposited energy from inelastic scattering was estimated using the same stopping-power expressions as those applied in the ionization analysis.

Assuming that incident electrons form a uniform heat source within an adiabatically confined spherical particle, the instantaneous temperature rise can be expressed by the Liu–Risbud model^[S2]^:

$\text{∆}\text{T}\text{= }\frac{\text{3}\text{jQ}}{\text{8}\text{e}\text{c}_{\text{v}}\text{D}_{\text{th}}\text{ρ}}\text{R}^{\text{2}}\text{ ln}\left( \text{1+ }\frac{\text{4}\text{D}_{\text{th}}\text{t}_{\text{e}}}{\text{R}^{\text{2}}} \right)$, (S-5)

where *j* is the electron current density, *Q* = *dE*/*dL* is the energy loss per electron, *e* is the elementary charge, *ρ* is the density, *c_v_* is the specific heat capacity at constant volume, *D*_th_ is the thermal diffusivity, *R* is the sphere radius, and *t_e_* is the dwell time.

Using the material parameters for WO_3_ (*ρ* = 6.28 g cm^-3^, *D*_th_ = 8.0 × 10^-7^ m^2^ s^-1^, *c_v_* = 323 J kg^-1^ K^-1^)^[S3,S4]^ and the STEM–ADF observation conditions shown in Figure 1(a,b) (*j* = 6.9 × 10^8^ A m^-2^, *t_e_* = 5 μs), the calculated temperature rise inside a 25-nm radius sphere reaches approximately 1500 K at 80 kV and 500 K at 300 kV. These values represent the upper limit under adiabatic confinement, neglecting axial heat dissipation.

Using this framework, we estimated the temperature rise of the nanowire. The total energy deposited into the nanowire by electron-beam irradiation (*E*_all_) can be expressed in terms of the heated volume *V*, density *ρ*, and constant-volume specific heat *c_v_* as

$\text{E}_{\text{all}}\text{ = ρ}\text{c}_{\text{v}}\text{V∆T }$. (S-6)

When the irradiation conditions are identical, *E*_all_ remains constant; thus, the temperature rise in a spherical particle and that in a nanowire can be related through their respective volume ratios. Denoting the temperature rise of a spherical particle as Δ*T*_particle_ and that of a nanowire under the same irradiation conditions as Δ*T*_wire_, we obtain

$\text{∆}\text{T}_{\text{wire}}\text{ =∆}\text{T}_{\text{particle}}\text{ }\frac{\text{V}_{\text{particle}}}{\text{V}_{\text{wire}}}$ . (S-7)

For a sphere of radius *R*, the volume is $\text{V}_{\text{particle}}\text{ =}\frac{\text{4}}{\text{3}}\text{ π}\text{R}^{\text{3}}$, whereas the effective heated volume of a nanowire with radius *R* is $\text{V}_{\text{wire}}\text{ = π}\text{R}^{\text{2}}\text{L}_{\text{eff}}$. Substituting these expressions yields

$\text{∆}\text{T}_{\text{wire}}\text{ =∆}\text{T}_{\text{particle}}\frac{\text{4}\text{R}}{\text{3}\text{L}_{\text{eff}}}$*.*  (S-8)

In a one-dimensional system with thermal diffusivity *D*_th_, the effective length over which heat spreads during an irradiation time *t_e_* is given by the thermal diffusion length $\sqrt{\text{2}\text{D}_{\text{th}}\text{t}_{\text{e}}}$. In this study, we approximate the effective heated length as $\text{L}_{\text{eff}}\text{ ≈ }\text{2}\sqrt{\text{2}\text{D}_{\text{th}}\text{t}_{\text{e}}}$. Substituting this into Eq. (S–8) gives the temperature rise within the nanowire as

$\text{∆}\text{T}_{\text{wire}}\text{ =∆}\text{T}_{\text{particle}}\text{ }\frac{\text{2}\text{R}}{\text{3}\sqrt{\text{2}\text{D}_{\text{th}}\text{t}_{\text{e}}}}$ . (S-9)

Thus, the temperature rise predicted by the adiabatic spherical model, Δ*T*_particle_, is reduced in the nanowire by the volume ratio $\frac{\text{4}\text{R}}{\text{3}\text{L}_{\text{eff}}}$ owing to heat diffusion along the nanowire axis. As a result, the effective temperature rise Δ*T*_wire_ decreases in proportion to the ratio between the radius *R* and the thermal diffusion length $\sqrt{\text{2}\text{D}_{\text{th}}\text{t}_{\text{e}}}$. Using this relationship, we estimated the temperature rise in the WO_3_ nanowire under electron-beam irradiation.

A cylindrical heat-diffusion model that accounts for axial heat conduction over the thermal diffusion length $\sqrt{\text{2}\text{D}_{\text{th}}\text{t}_{\text{e}}} \approx$ 2.8 μm was solved under steady-state conditions. The calculated temperature rise under typical STEM–ADF imaging conditions (*j* = 6.9 × 10^8^ A m^-2^, *t_e_* = 5 μs) was approximately 9 K at 80 kV and 3 K at 300 kV, indicating efficient thermal dissipation along the nanowire.

**References**

[S1] D.C. Joy, and S. Luo (1989) An empirical stopping power relationship for low-energy electrons. *Scanning* 11: 176-180. <https://doi.org/10.1002/sca.4950110404>

[S2] A. Kryshtal, M. Mielczarek, and J. Pawlak (2022) Effect of electron beam irradiation on the temperature of single AuGe nanoparticles in a TEM. *Ultramicroscopy* 233: 113459. <https://doi.org/10.1016/j.ultramic.2021.113459>

[S3] H. Wang, Y. Xu, M. Goto, Y. Tanaka, M. Yamazaki, A. Kasahara, and M. Tosa (2006) Thermal Conductivity Measurement of Tungsten Oxide Nanoscale Thin Films. *Mater. Trans.* 47: 1894-1897. <https://doi.org/10.2320/matertrans.47.1894>

[S4] H. Seltz, F.J. Dunkerley, and B.J. Dewitt (1943) Heat Capacities and Entropies of Molybdenum and Tungsten Trioxides. *J. Am. Chem. Soc.* 65: 600-602. <https://doi.org/10.1021/ja01244a030>
